# The Evolutionary Dynamics of Mechanically Complex Systems

**DOI:** 10.1093/icb/icz077

**Published:** 2019-05-27

**Authors:** Martha M Muñoz

**Affiliations:** Department of Biological Sciences, Virginia Tech, Blacksburg, VA 24060, USA

## Abstract

Animals use a diverse array of motion to feed, escape predators, and reproduce. Linking morphology, performance, and fitness is a foundational paradigm in organismal biology and evolution. Yet, the influence of mechanical relationships on evolutionary diversity remains unresolved. Here, I focus on the many-to-one mapping of form to function, a widespread, emergent property of many mechanical systems in nature, and discuss how mechanical redundancy influences the tempo and mode of phenotypic evolution. By supplying many possible morphological pathways for functional adaptation, many-to-one mapping can release morphology from selection on performance. Consequently, many-to-one mapping decouples morphological and functional diversification. In fish, for example, parallel morphological evolution is weaker for traits that contribute to mechanically redundant motions, like suction feeding performance, than for systems with one-to-one form–function relationships, like lower jaw lever ratios. As mechanical complexity increases, historical factors play a stronger role in shaping evolutionary trajectories. Many-to-one mapping, however, does not always result in equal freedom of morphological evolution. The kinematics of complex systems can often be reduced to variation in a few traits of high mechanical effect. In various different four-bar linkage systems, for example, mechanical output (kinematic transmission) is highly sensitive to size variation in one or two links, and insensitive to variation in the others. In four-bar linkage systems, faster rates of evolution are biased to traits of high mechanical effect. Mechanical sensitivity also results in stronger parallel evolution—evolutionary transitions in mechanical output are coupled with transition in linkages of high mechanical effect. In other words, the evolutionary dynamics of complex systems can actually approximate that of simpler, one-to-one systems when mechanical sensitivity is strong. When examined in a macroevolutionary framework, the same mechanical system may experience distinct selective pressures in different groups of organisms. For example, performance tradeoffs are stronger for organisms that use the same mechanical structure for more functions. In general, stronger performance tradeoffs result in less phenotypic diversity in the system and, sometimes, a slower rate of evolution. These macroevolutionary trends can contribute to unevenness in functional and lineage diversity across the tree of life. Finally, I discuss how the evolution of mechanical systems informs our understanding of the relative roles of determinism and contingency in evolution.

## Introduction

 A fundamental property of biological systems is that diversity is unequally distributed: whereas some traits, and lineages achieve evolutionary overdrive, other appear to stall over long periods of time (Schluter 2000; Gingerich 2009). But, why is this true? What intrinsic and extrinsic variables facilitate evolution in some cases and cause it to stall in others? For centuries, the concept of constraint and release from constraint has been a cornerstone for biology (Goethe 1823; Owen 1847, 1848; Thompson 1917). This topic centers around the idea of “evolvability” by asking which organismal and environmental features tend to expand or limit phenotypic variation, and which factors tend to accelerate or slow evolutionary change (Parker and Maynard Smith 1990; Wagner and Altenberg 1996; Gould 2002; Futuyma 2010).

Central to this debate is organismal structure and the physical relationships guiding biological motion (Olson and Miller 1958; Lauder 1981). Mechanical structures reflect inherent tradeoffs and relationships that bound the limits of morphospace and shape the peaks and valleys of the performance landscape (Raup 1966; Seilacher 1970; Arnold 2003; Polly 2008). The performance landscape describes how morphological trait combinations map to performance (Arnold 1983), which can help conceptualize the evolutionary consequences of form-function variation. For example, some features of organismal design, such as key innovations, can drastically re-organize the landscape and impact rates of trait evolution (Liem 1973; Vermeij 1999). Thus, to deeply understand how diversity is distributed across the tree of life, the structural properties of organisms must be explicitly considered in an evolutionary framework (Seilacher 1970; Lauder 1981; Wake 1982; Gould 2002).

The relationships between mechanical and evolutionary diversity can be conceptualized through the performance landscape, which reflects how trait variation contributes to ecologically-relevant functions (e.g., Arnold 1983, 2003). Mechanics shapes the performance landscape by reflecting how organisms can physically interact with their environments. Consider, for example, a simple lever system like a mammalian lower jaw or a crustacean claw. The structure’s closing force is a function of the lever’s mechanical advantage, which is determined by the relative proportions of the lever arms on either side of the fulcrum (Elner and Campbell 1981; Liem et al. 2001). Trade-offs between force and speed and contribution from other components (such as surrounding muscles) are reflected in the performance landscape for lever systems (Westneat 2004; Levinton and Allen 2005; Slater and Van Valkenburgh 2009; Grossnickle 2017). In turn, the mechanical performance of the levers is filtered through a fitness gradient that is shaped by the ecological context in which organisms express their phenotypes (Garland and Losos 1994). For example, the presence of new prey types or predators might alter the presence, location, and shape of adaptive performance peaks.

Thus, the performance landscape provides a clear conceptual bridge between morphological variation and organismal fitness in biomechanical systems. One of the most commonly studied properties of mechanical systems is the many-to-one mapping of morphology to performance (Wainwright et al. 2005; Wainwright 2007). In mechanical systems comprised of three or more parts, multiple morphological pathways can produce common performance outputs. For example, the ability for fish to generate suction force during feeding reflects a combination of several skeletal and muscular features that contribute to buccal expansion (Collar and Wainwright 2006; Holzman et al. 2008). Different configurations of these features can result in similar suction forces. In recent years, there has been a growing appreciation that many-to-one mapping is ubiquitous in nature and significantly contributes to patterns of evolutionary diversity (Wainwright 2007). But, how does many-to-one mapping actually impact the evolutionary dynamics of mechanical systems? Does redundancy impact how rapidly diversity can accumulate, and can it impact the total phenotypic disparity that a mechanical system exhibits (e.g., Sidlauskas 2008)?

In this paper, I describe how many-to-one mapping influences the distribution of phenotypic diversity by considering two key aspects of trait evolution: tempo (the rate at which disparity accumulates) and mode (the pattern of trait variation). I also describe how mechanical sensitivity—differential relationships among parts within a mechanical system—guides the evolution of functionally redundant systems. Finally, I consider how distinct selective pressures among different groups of organisms can impact the rate and pattern of complex mechanical systems. Although I provide examples using mechanically redundant systems, the general principles and approaches can be applied to a wide variety of systems and at multiple scales of analysis. The goal of this paper is to conceptually unify a disparate literature through common themes of functional constraint and evolutionary diversity.

### Rates of morphological evolution in mechanically redundant systems

By providing multiple morphological pathways to similar mechanical outputs, many-to-one mapping can decouple morphological and performance evolution (Alfaro et al. 2004, 2005; Collar and Wainwright 2006). Suction-feeding in teleost fishes provides a key example of this phenomenon. Expansion of the buccal cavity during feeding creates subambient pressure that draws water (and food) into the fish’s mouth (Van Leeuwen and Muller 1983; Lauder and Clark 1984). Suction-feeding performance (acceleration and velocity of water into the mouth) involves coordination among multiple skeletomuscular traits (Muller et al. 1982; Carroll et al. 2004; Higham et al. 2006; Wainwright et al. 2007). Different size combinations of these traits—for example, gape width, buccal length, cross-sectional area of the epaxial muscle, lengths of the epaxialis, and buccal cavity moment arms—can result in similar suction forces, indicating many-to-one mapping of form to function (Carroll et al. 2004; Wainwright et al. 2007). Importantly, functional decoupling is associated with evolutionary decoupling of morphological and mechanical diversity. For example, in a study of sunfishes (Family: Centrarchidae), Collar and Wainwright (2006) found a weak association between rates of morphological and suction performance evolution. These results indicate that strong selection on suction feeding (or any other performance metric) in nature need not be matched with equally strong selection on morphological traits. Specifically, by diffusing the pathways to functional change among several traits, many-to-one mapping can effectively dampen selection for large changes in morphological evolution.

A corollary of these findings is that many-to-one mapping should impact patterns of morphological evolution (Wainwright et al. 2005; Wainwright 2007). Because multiple morphological combinations can yield a common mechanical output, it might be reasoned that all traits in a complex system should be equally likely to evolve in response to selection on performance. It turns out, however, that this is not always true. The functional output of a mechanical system is often disproportionately sensitive to variation in some components relative to others, a phenomenon known as “mechanical sensitivity” (Anderson and Patek 2015). Whereas small changes in one component may have a large impact on mechanical output, relatively large changes in a different component may have only a negligible impact on the system’s motion (Koehl 1996). In other words, changes in one or a few traits are sufficient to produce a given performance output. As such, some morphological traits in complex functional systems might be freer to vary than others.

**Fig. 1:**
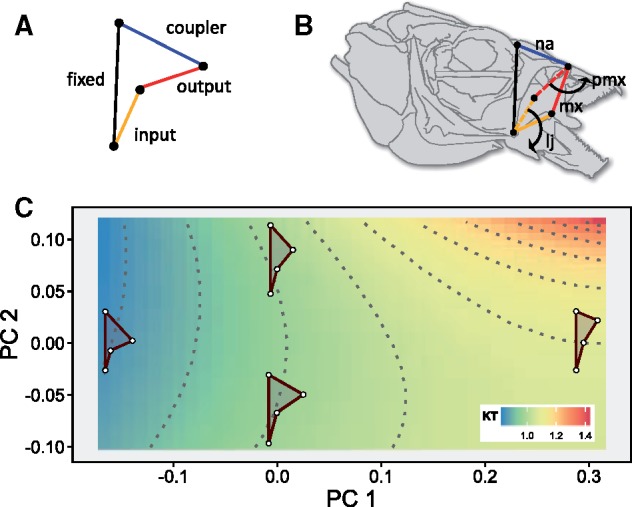
Four-bar linkages are a key example of many-to-one mapping in nature. (**A**) Four-bar linkages consist of a fixed link (black) and three mobile links: input (orange), output (red), and coupler (blue). (**B**) In an example teleost oral four-bar system, the input link (lower jaw, lj) rotates ventrally, causing rotation in the nasal (na) and in the output link (maxilla, mx), resulting in premaxillary (pmx) protrusion. (**C**) Many-to-one mapping of four-bar morphospace to KT. The principal components of oral four-bar phylomorphospace are plotted simultaneously with contours of maxillary KT. Isoclines of KT show morphological combinations with equivalent maxillary KT values. The data shown are from Malagasy cichlids (Martinez and Sparks 2017), and the color contour map was provided by C. Martinez. Image from 1B is reproduced from Muñoz et al. (2018).

Four-bar linkages provide the best documented example of mechanical sensitivity in nature (Anderson and Patek 2015; Hu et al. 2017; Muñoz et al. 2017, 2018). Four-bar linkages are closed-chain mechanical systems comprised of four rigid levers (links) that interact to transmit force and motion (Anker 1974; Westneat 1990; De Visser and Barel 1996; Patek et al. 2007) (Fig. 1A). Three of those links (input, output, and coupler) are mobile and rotate relative to a fourth, fixed link. Four-bar linkages are widespread in nature, enabling such diverse motions as upper and lower jaw protrusion in fishes, rapid raptorial strikes in mantis shrimp, and cranial kinesis in birds (Hulsey and Wainwright 2002; Wainwright et al. 2005; Patek et al. 2007; Olsen and Westneat 2016). The motion of four-bar linkages has been most often characterized using kinematic transmission (KT), which describes the amount of input rotation relative to output rotation in the system. All else being equal, KT reflects a tradeoff between displacement and force. There are many different link size combinations that can produce similar KT values (Fig. 1C); thus, four-bar linkages are mechanically redundant structures. There are a few limitations to comparing four-bar linkages among groups of animals. First, KT is dynamic in that it changes non-linearly during rotation (Patek et al. 2007). Consistency in linkage rotation among systems and/or estimates of minimum KT during rotation can partially overcome this limitation (Wainwright et al. 2005; Muñoz et al. 2018). Second, ratio-based metrics (such as KT) are limited perspectives on mechanical equivalency when comparing across different groups of organisms (e.g., Cooper and Westneat 2009). However, when used as a biological heuristic within a group of similar organisms, ratios like KT provide a useful perspective on four-bar linkage motion along the force-displacement continuum. For example, if selection were to favor a transition to molluscivory in a group of fish, we would expect the oral four-bar linkage to shift toward more force-based motion (e.g., toward the blue region of Fig. 1C) and, due to mechanical redundancy, there are several potential pathways by which this could be accomplished (Wainwright et al. 2004; Hulsey and García De León 2005).

**Fig. 2:**
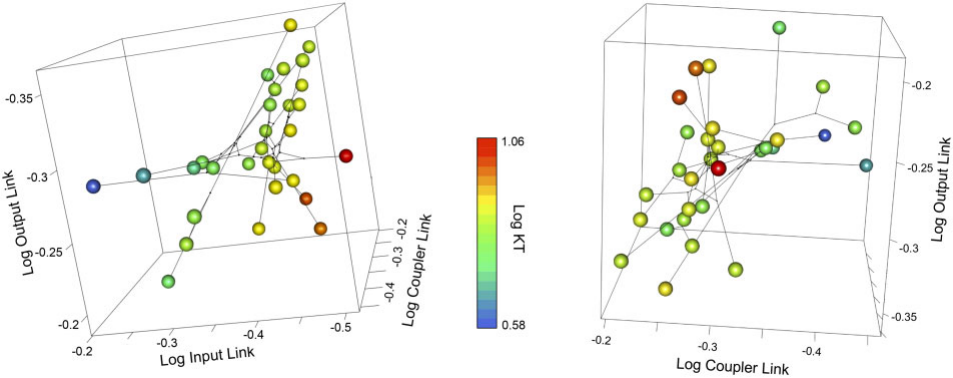
Mechanical sensitivity in the cichlid oral four-bar linkage system. Each plot is a 3D phylomorphospace showing variation in input link, output link, and coupler link length for 30 species of cichlid fish. Each point in the plots corresponds to a different cichlid species. The color of each point corresponds to the estimated maxillary KT of that species’ oral four-bar linkage. In the left panel, the strong mechanical sensitivity of maxillary KT to input link size is illustrated (note color gradient from blue to red). In the right panel, the lack of sensitivity with regard to the output and coupler links is illustrated (no color gradient). Original data: Hulsey and García De León 2005. Phylogeny: Hulsey et al. 2010. Phylomorphospace plots: Muñoz et al. 2018, supp. mat.

The motion of four-bar linkages is often differentially sensitive to link size shifts: varying some link sizes results in little net change, while varying other links even a little drastically alter mechanical output (Anderson and Patek 2015; Hu et al. 2017; Muñoz et al. 2018). For example, in the cichlid oral-four bar system, KT is strongly positively correlated with input link length (i.e., high sensitivity), and much less correlated with coupler and output link length (i.e., low sensitivity) (Fig. 2). In other words, a small change in the size of the input link results in a disproportionately larger effect on the motion of the system than an equivalent change in the output link of the system. Patterns of mechanical sensitivity differ among systems. For example, in several other four-bar systems (the mantis shrimp raptorial four-bar, the sunfish opercular four-bar, and the wrasse oral four-bar), KT is much more sensitive to variation in the output link than to variation in the input or coupler links of the system (Muñoz et al. 2018). The proximate mechanism for this pattern appears to be size: even slight changes to relatively small linkages will disproportionately impact four-bar geometry and motion (Muller 1996).

The key point is that mechanical redundancy (many morphological pathways to a single performance) is not tantamount to morphological equivalency (equal functional consequences for a given morphological change) (Anderson and Patek 2015; Hu et al. 2017; Baumgart and Anderson 2018). But, why should this matter? This distinction is important because rates of morphological evolution can vary based on differences in mechanical sensitivity. In each of the four-bar systems described above, mechanical sensitivity (a stronger correlation between link length and KT) was consistently associated with faster rates of trait evolution (Muñoz et al. 2018). Furthermore, mechanical sensitivity and evolutionary rate disparity were both tightly associated with relative size: when a certain linkage was particularly small, mechanical sensitivity was especially strong because even small changes in size more dramatically alter four-bar geometry (Muller 1996), and rates of evolution for very small links were especially rapid (Muñoz et al. 2018). Whereas many-to-one mapping theoretically provides multiple potential pathways for morphological adaptation (as illustrated by the contours of performance space in Fig. 1C), mechanical sensitivity determines which of these are more likely to evolve. Or, put differently, many-to-one mapping does not imply equal freedom of evolution among morphological traits.

This point—that rates of trait evolution are unequal in mechanically redundant systems—is subtle but important because it could belie different selective mechanisms among morphological traits that are, as yet, unclear. Evolutionary shifts in functional systems can often be attributed to differences in ecological selective pressures (Martin and Wainwright 2011; Martin 2016). Is evolutionary rate disparity due to directional selection on links of high mechanical effect, due to stronger stabilizing selection on other linkages, or some combination of both? More broadly, are some morphological elements under stronger genetic constraints and others more able to evolve independently (Wagner and Altenberg 1996; Albertson et al. 2005)? Simply put, comparing rates of trait evolution can reveal patterns that are conceptually linked to structural constraints (mechanical sensitivity), but we cannot yet discern how mosaic morphological evolution reflects the selective environments to which mechanical systems respond.

### Patterns of morphological evolution in mechanically redundant systems

**Fig. 3:**
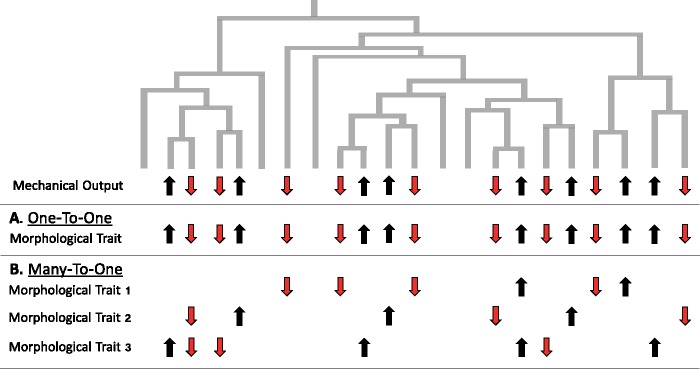
Many-to-one mapping should weaken parallel morphological evolution. A hypothetical phylogeny showing evolutionary shifts in mechanical output on different branches. In some cases, mechanical output increases (black arrows) and in other cases mechanical output decreases (red arrows). (**A**) If the relationship between morphology and mechanical output is one-to-one, evolutionary shifts in performance are expected to be associated with shifts in morphology. For simplicity, the relationship between trait size and mechanical output are assumed to be positive. Thus, one-to-one form–function relationships should result in strong parallel evolution. (**B**) If the relationship between morphology and mechanical output is many-to-one, then evolutionary shifts in performance could be accompanied by shifts in any combination of morphological traits. For example, an increase in output could be accompanied by a shift in Trait 1, Trait 2, Trait3, or any combination of those traits. For simplicity, the relationship between all traits and mechanical output is assumed to be positive. Note that, because the hypothesized scenario above focuses on closely related species, I have opted to use “parallelism” to describe similar evolutionary outcomes in morphology, but “convergence” is also an applicable term.

In addition to impacting evolutionary rates, many-to-one mapping should affect phylogenetic patterns of trait variation. By definition, many-to-one mapping provides multiple morphological pathways for functional adaptation (Wainwright et al. 2005, Wainwright 2007). Consequently, selection for certain functional demands—such as greater suction force in different populations of fishes—might not be met by the same morphological “solutions” (Wainwright 2007). As a corollary, similar selection on function (e.g., across multiple populations) might not be associated with similar patterns of morphological evolution (McGee and Wainwright 2013; Langerhans 2017). In contrast, one-to-one mapping canalizes the morphological pathways to adaptation, so similar patterns of selection should result in similar evolutionary patterns. Thus, a consequence of mechanical redundancy should be weaker evolutionary convergence (or parallelism if the starting conditions are similar, such as different populations of the same species) (Fig. 3), implying that the morphological pattern of evolution should become less predictable in more functionally complex systems.

This prediction is remarkably well-supported among populations of the threespine stickleback fish (*Gasterosteus aculeatus*) from lake and stream environments (Thompson et al. 2017). Trophic ecology differs markedly between lake and stream forms, with lake stickleback incorporating more limnetic prey and exhibiting a higher trophic position than stream stickleback, which tend to incorporate more benthic prey (Kaeuffer et al. 2012). These trophic differences are important because selection on diet is often associated with strong selection on the feeding apparatus (e.g., Martin and Wainwright 2011). Indeed, across 16 replicate pairs of lake and stream stickleback populations, transitions to a different environment were associated with a strong shift in feeding biomechanics. These included shifts in mechanically simple traits like KT of the lower jaw (estimated as the ratio of jaw out- and inlever lengths), as well as more complex mechanical traits, such as KT of the oral four-bar linkage system and suction index. As mechanical complexity increased, the specific morphological pathway for functional adaptation became progressively less predictable (i.e., weaker parallelism, Thompson et al. 2017).

The signatures of mechanical redundancy on parallel evolution can also manifest at deeper phylogenetic scales. Leaf-nosed bats (family Phyllostomatidae) exhibit exceptional diversity among mammals for diet, ranging from fully liquid diets such as blood to hard materials such as bone (Dumont 1999; Cruz-Neto et al. 2001), and many switches in diet occurred throughout bat evolutionary history (Santana and Dumont 2009; Santana et al. 2012). Biting hard materials has strong functional demands, and requires translating muscle force into a stronger bite force (Santana et al. 2010). Correspondingly, phylogenetic shifts in bats to harder prey were consistently associated with the evolution of larger temporalis muscles (Santana et al. 2012). In contrast, specialization to more liquid-based diets exhibited many-to-one mapping of form to function. The morphological pathway accompanying a dietary transition to a liquid diet was less predictable among species (Santana et al. 2012). Thus, both within species and across species, parallelism in the pattern of morphological evolution becomes weaker in progressively more complex functional systems: as the number of morphological pathways to adaptation for a particular function increase, other factors, like drift and historical contingency, are likely to play a strong role in structuring patterns of morphological evolution (Walker 2007).

Due to mechanical sensitivity, however, not all evolutionary pathways in functionally redundant systems may be equally likely to evolve. Mechanical sensitivity should bias evolutionary shifts to traits of high mechanical effect. Traits of high mechanical effect approximate a one-to-one form–function relationship (even in complex systems); thus, mechanical sensitivity is expected to result in stronger parallel evolution (i.e., approximate the pattern in Fig. 3A despite multiple traits). For example, in the wrasse oral four-bar linkage system, KT was highly sensitive to variation in the output and input links and relatively insensitive to variation in the coupler link (Muñoz et al. 2018). Evolutionary shifts in KT across the wrasse tree were always accompanied by a concomitant shift in the input and output links, whereas none were detected in the coupler link (Muñoz et al. 2018). In other words, mechanical sensitivity restricts the number of morphological “solutions,” resulting in a more predictable pattern of evolution despite a greater number of potential pathways. Nonetheless, among mechanically sensitive links, there was equal freedom of evolution (i.e., shifts were equally likely to include the output or input link), illustrating that evolution does become less parallel when potential pathways are, in fact, mechanically equivalent.

The central point is that, while many-to-one mapping theoretically limits the predictability of trait evolution, mechanical sensitivity can serve to constrain evolutionary pathways to traits of high mechanical effect. Depending on the strength of mechanical sensitivity, parallelism should be very weak (when sensitivity is equivalent or nearly so among traits) or very strong (when sensitivity is highly biased to a single trait). The not-so-parallel evolution of mechanical systems raises several key questions about evolutionary predictability. For example, many other potential mechanisms (besides mechanical sensitivity) may determine which morphological feature of a complex system will shift, such as genetic correlations, developmental constraints, and mutation order (Herron and Doebeli 2013; Bright et al. 2016). Are patterns of evolution ultimately predictable if we integrate information across different scale of organizations, or are mechanically equivalent pathways truly equally likely to evolve?

### The macroevolutionary dynamics of mechanical systems

A key lens with which to consider the evolution of mechanical structures and functional performance is in the broader context of lineage diversification (e.g., Price et al. 2010, 2012). The major theme of this section is that the tempo and mode of mechanical evolution varies among groups of organisms. For example, although geckos and anoles have independently evolved adhesive toepads, the adaptive landscape varies substantially among these two groups (Hagey et al. 2017). In anoles, adhesive performance evolves in a more bounded fashion (i.e., reflecting a more phenotypically restricted adaptive zone), whereas in geckos, adhesive performance appears to explore a wider range of adaptive zones, reflected by less phenotypically bounded evolution (Hagey et al. 2017).

Why should patterns of mechanical evolution differ among groups of organisms? One of the key reasons is that they might experience a different range of selective pressures. For example, some biomechanical structures are involved in many different functions in some organisms, whereas in others they could be involved in fewer (Moen 2019). Theoretically, multi-functionality could be an impediment to evolution because tradeoffs from multiple completing performance demands should preclude adaptive change (Walker 2007). Alternatively, however, multi-functionality may not limit phenotypic adaptation; in some cases, it could result in broader phenotypic variation or even in a faster rate of evolution (e.g., Holzman et al. 2012; Shoval et al. 2012).

Turtles provide an interesting system in which to compare these two possibilities because aquatic and terrestrial species differ in the number of performance demands that their shells experience. For example, whereas both aquatic and terrestrial turtles must resist stress, only aquatic turtle shells must also be hydrodynamic (i.e., reduce drag during swimming) (Claude et al. 2003; Rivera 2008). In a comparison of terrestrial and aquatic turtles, Stayton (2011) found that multiple regions of shell morphospace resulted in highly load-resistant turtle shells, indicating many-to-one mapping of form to function in both aquatic and terrestrial species. Nonetheless, there were fewer morphological combinations that could simultaneously optimize both load resistance and reduce drag, suggesting that greater performance tradeoffs in aquatic species should limit their phenotypic evolution (Stayton 2011; see also Moen 2019 for a similar example in frog locomotor performance). Correspondingly, terrestrial turtles consistently exhibit greater phenotypic diversity than aquatic species (Stayton et al. 2018). Interestingly, lower phenotypic diversity in aquatic turtle shells was not associated with a slower evolutionary rate (Stayton et al. 2018). This result echoes other studies that find that greater exploration of morphospace is not necessarily accompanied by a faster rate of morphological change, which illustrates that evolutionary tempo and mode can be decoupled (e.g., Sidlauskas 2008).

Similarly, transitions from suction-based feeding (requiring complex mechanical coordination among several features) to biting-based feeding (requiring less coordination) in eels was accompanied by phenotypic expansion into a wider range of morphospace, but not with any shifts in evolutionary rates (Collar et al. 2014). Thus, the finding that the tempo and mode of evolution in complex structures can be decoupled may be a general feature of biomechanical evolution. The findings from turtles and eels suggest that stronger performance tradeoffs may not impact evolutionary rate, but this result is far from universal. For example, stronger biomechanical tradeoffs in feeding performance in fish are associated with faster rates of evolution (Holzman et al. 2012). Even in this case, however, the strength of the associated varied substantially among different fish lineages (Holzman et al. 2012). Thus, extending performance tradeoffs to a more general evolutionary framework is challenging.

Functional systems may experience the same number of selective pressures, but the strength of those pressures might vary among lineages. The four-bar linkage of the mantis shrimp raptorial appendage (Patek et al. 2004, 2007; Anderson et al. 2014) provides a clear example of this phenomenon. Mantis shrimp use their four-bar linkage system to capture prey in one of two main ways. Whereas “spearers” use their pointed appendage to ensnare elusive, soft-bodied prey such as fish (these emphasize greater displacement, higher KT), “smashers” possess a club-like appendage that they use to bludgeon hard-shelled prey, such as snails (these emphasize greater force, lower KT) (Patek and Caldwell 2005; Patek et al. 2007; Patek 2015). In the mantis shrimp raptorial four-bar, KT is highly sensitive to variation in the output link; correspondingly, this link evolves much more rapidly than the input and coupler links (Anderson and Patek 2015; Muñoz et al. 2017). But output link evolution proceeds unequally among types of mantis shrimp. Perhaps due to stronger functional constraints associated with producing large impact forces, the rate of output link evolution is substantially slower in smashers than in spearers, a result that is echoed in other morphological aspects of the raptorial appendage (Claverie and Patek 2013; Muñoz et al. 2017). Spearers, in contrast, may not need to produce ultrafast strikes to snag elusive prey (de Vries et al. 2012; McHenry et al. 2012). In the context of the adaptive landscape, these results suggest that the forces required to successfully break through shells might result in a narrower adaptive peak for performance, whereas spearers might be able to “explore” a broader range of functional phenotypes about a relatively wider adaptive peak.

As illustrated above, lineage-specific differences in the adaptive landscape can directly impact the observed tempo and mode of form–function evolution. Shifts in the adaptive landscape may reflect changes in selective pressures (Arbour and López-Fernández 2016), which could contribute substantially to major patterns of lineage heterogeneity across the tree of life (e.g., Alfaro et al. 2009; Near et al. 2013; Cooney et al. 2017). For example, by expanding the number of morphological “solutions” to a shared ecological pressure, many-to-one mapping can serve to increase morphological diversity (Wainwright 2007; McGee and Wainwright 2013). Explicitly incorporating the performance landscape into macroevolutionary studies requires especially rich phylogenetic, morphological, and mechanical datasets: in the new age of big data in biology, such goals are becoming increasingly tractable (e.g., Tendler et al. 2015; Davies et al. 2017; Dickson and Pierce 2019; Stayton 2019).

### Concluding remarks

A long-standing question in biology is whether evolution should be considered highly predictable or, instead, contingent on chance events (discussed in Losos 2017; Blount et al. 2018). Stephen Jay Gould famously proposed a thought experiment: if one were to go back in evolutionary history and “replay the tape of life,” would the evolutionary outcomes be similar or different (Gould 1989)? Is evolution highly deterministic, resulting in similar evolutionary outcomes across different replays, or is evolution more subject to the vagaries of historical contingency? Similar mechanical systems in nature can be considered independent evolutionary experiments or different “replays” of the tape of life. For example, powered flight, suction-based feeding, and four-bar linkages have independently evolved in several animal systems. As evidenced by repeatable patterns from the level of populations to whole lineages, many aspects of form–function evolution are highly deterministic. When form–function relationships exhibit one-to-one mapping, selection often finds the same solutions to similar problems. Even in systems exhibiting many-to-one mapping, mechanical sensitivity can bias morphological evolution to a few traits of high mechanical effect. Similarly, when mechanical systems are subject to strong performance tradeoffs, phenotypic diversity is lower than in systems that experience weaker tradeoffs.

Different replays of similar mechanical systems appear to often converge on similar evolutionary outcomes. However, in systems exhibiting many-to-one mapping, morphological evolution becomes substantially less predictable, particularly when morphological solutions to functional demands are truly mechanically equivalent (i.e., similar mechanical sensitivity). Moreover, whether stronger performance tradeoffs should result in a shift in evolutionary rate is unclear, as the pattern varies substantially among systems. Thus, in several cases, the nuance of history may play a stronger role in structuring evolution. Perhaps a minor “jot or tittle” (Gould 1989) in a lineage’s history can dramatically alter evolutionary trajectories in mechanically redundant systems. Then again, there are certainly additional layers of constraint (such as genetics and development) sculpting evolution that are not evident from the scale of whole-organism mechanical systems.

Evolutionary studies of mechanical systems are revealing repeatable micro- and macroevolutionary patterns, and also revealing where those patterns tend to fall apart. Moving forward, these concepts can be treated as hypothesis frameworks to be rigorously tested across distinct temporal, spatial, and phylogenetic scales. Despite much progress into the macroevolutionary dynamics of biomechanical systems over the past several decades, there is much still to be discovered. For example, how do form–function relationships scale from individuals to populations and species? Do similar mechanical structures predictably result in convergent patterns of morphological evolution across distantly related organisms? There are two key lenses with which future studies might tackle these (and other) questions. On the one hand, researchers might start at the macroevolutionary level to identify species- or clade-level shifts in morphology or performance. Such a “phylogenetic natural history” approach emphasizes the perspective that broadscale data provide for generating testable hypotheses of mechanism at shallower scales (Uyeda et al. 2018). On the other hand, researchers might opt to use form–function relationships (e.g., performance tradeoffs) to predict micro- and macroevolutionary patterns. Top-down and bottom-up approaches provide equally important and complementary perspectives on biomechanical evolution.

## Notes

From the symposium “Comparative Evolutionary Morphology and Biomechanics in the Era of Big Data” presented at the annual meeting of the Society for Integrative and Comparative Biology, January 3–7, 2019 at Tampa, Florida.
